# Supplementary material for: Nutrient Digestive Bypass: Determinants and Associations with Stool Quality in Cats and Dogs
Source: Animals (Basel). 2024 Sep 26;14(19):2778. doi: 10.3390/ani14192778 (PMC11475581; doi:10.3390/ani14192778)
Supplement: Supplementary file 1 [file animals-14-02778-s001.zip › animals-3117894-supplementary.pdf]

Supplementary Materials

Table S1. Huber robust outlier rejection application and changes on body wt (kg) and digestibility (%) endpoints.

Species x Product Type	Endpoint	Initial			Final			% Center Change after Outlier Removel
		Huber Center	Huber Spread	Huber N Outliers	Huber Center	Huber Spread	Huber N Outliers	
Canine Dry	(final BW−Initial BW)/Initial BW × 100	−0.09	1.98	31	−0.06	1.93	0	30.3%[α]
	Apparent Carbohydrate Digestibility	89.13	5.46	47	89.23	5.18	0	0.1%
	Apparent Dry Matter Digestibility	83.63	6.62	10	83.93	6.27	0	0.4%
	Apparent Energy Digestibility	86.53	5.98	34	86.89	5.55	0	0.4%
	Apparent Fat Digestibility	93.90	2.56	72	94.05	2.39	0	0.2%
	Apparent Fiber Digestibility	32.13	20.46	42	33.06	19.59	0	2.9%
	True Protein Digestibility	89.81	4.68	26	89.96	4.51	0	0.2%
Canine Wet	(final BW−Initial BW)/Initial BW × 100	−0.43	2.10	10	−0.41	2.00	0	4.9% [α]
	Apparent Carbohydrate Digestibility	88.69	4.75	42	89.37	3.98	0	0.8%
	Apparent Dry Matter Digestibility	83.35	5.73	9	84.29	4.74	0	1.1%
	Apparent Energy Digestibility	85.33	5.37	21	86.09	4.55	0	0.9%
	Apparent Fat Digestibility	92.06	4.62	13	92.57	4.13	0	0.6%
	Apparent Fiber Digestibility	39.81	22.71	2	41.59	21.81	0	4.5%
	True Protein Digestibility	87.64	5.26	2	88.03	4.86	0	0.5%
Feline Dry	(final BW−Initial BW)/Initial BW × 100	0.48	2.18	27	0.48	2.12	0	0.3% [α]
	Apparent Carbohydrate Digestibility	87.14	5.88	23	87.34	5.65	0	0.2%
	Apparent Dry Matter Digestibility	83.51	5.55	8	83.73	5.35	0	0.3%
	Apparent Energy Digestibility	86.52	4.95	21	86.73	4.74	0	0.2%
	Apparent Fat Digestibility	92.13	3.25	31	92.23	3.15	0	0.1%
	Apparent Fiber Digestibility	30.73	25.51	8	30.97	25.28	0	0.8%
	True Protein Digestibility	93.44	4.78	6	93.55	4.68	0	0.1%
Feline Wet	(final BW−Initial BW)/Initial BW × 100	−1.58	2.52	16	−1.43	2.39	0	9.6% [α]
	Apparent Carbohydrate Digestibility	82.43	9.81	17	84.28	7.75	0	2.2%
	Apparent Dry Matter Digestibility	81.14	6.27	5	82.30	5.11	0	1.4%
	Apparent Energy Digestibility	83.34	6.32	3	84.50	5.13	0	1.4%
	Apparent Fat Digestibility	88.72	5.76	25	89.61	4.77	0	1.0%
	Apparent Fiber Digestibility	31.87	25.72	10	32.54	25.71	0	2.1%
	True Protein Digestibility	93.26	4.81	1	93.73	4.65	0	0.5%

[α]These means are numerically elevated as the foods are fed with the intention of maintaining body weight. Therefore, with an initial denominator which is approaching zero is used as a percent change the reported percent center change can be high without a significant change in actual body weight change over time.

Table S2. Numeric values of number of data points, tests, foods and pets used.

**Data Points**								
total	dog	cat	dry	wet	dogDry	dogWet	catDry	catWet
11271	5789	5482	8253	3018	4525	1264	3728	1754
**Tests**								
total	dog	cat	dry	wet	dogDry	dogWet	catDry	catWet
2020	1028	992	1446	574	798	230	648	344
**Foods**								
total	dog	cat	dry	wet	dogDry	dogWet	catDry	catWet
1885	966	919	1337	548	744	222	593	326
**pets**								
dog				cat				
361				536				

Table S3. The effect of crude fiber intake and fecal fiber on digestibility and fecal protein and fat.4556

Independent Var	Dependent Variable	Term	Canine					Feline				
			Prob>|t|	Estimate	Std Error	95% Lower	95% Upper	Prob>|t|	Estimate	Std Error	95% Lower	95% Upper
Fiber Intake	Protein TTTD	Intercept	0.01	90.985	1.021	78.712	103.257	<0.0001	93.627	0.203	93.057	94.196
Fiber Intake	Protein TTTD	Fiber	<0.0001	−16.279	0.627	−17.509	−15.049	<0.0001	−20.978	1.845	−24.594	−17.362
Fiber Intake	Fat TTTD	Intercept	<0.0001	99.091	0.714	90.615	107.567	0.006	97.892	1.005	85.763	110.022
Fiber Intake	Fat TTTD	Fiber	<0.0001	−4.686	0.335	−5.343	−4.028	<0.0001	−16.318	1.543	−19.343	−13.293
Fecal Fiber	Fecal Protein	Intercept	0.026	1.114	0.056	0.513	1.715	<0.0001	0.718	0.018	0.649	0.787
Fecal Fiber	Fecal Protein	Fiber	<0.0001	0.147	4.0E−03	0.139	0.155	<0.0001	0.208	0.007	0.193	0.222
Fecal Fiber	Fecal Fat	Intercept	0.075	0.319	0.039	−0.156	0.794	0.088	0.279	0.040	−0.209	0.767
Fecal Fiber	Fecal Fat	Fiber	<0.0001	0.024	1.1E−03	0.022	0.026	<0.0001	0.036	0.003	0.030	0.042

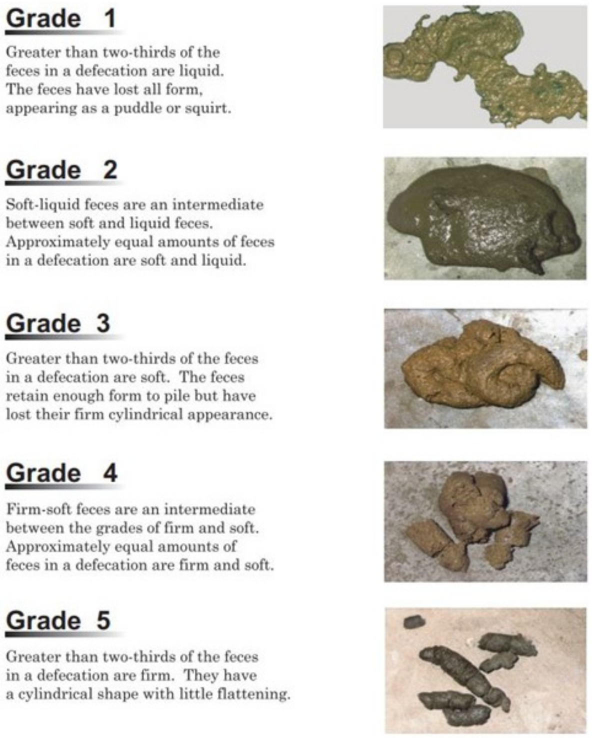

Figure S1. Description and guide to stool grading
